# Bioprospecting for plant resilience to climate change: mycorrhizal symbionts of European and American beachgrass (*Ammophila arenaria* and *Ammophila breviligulata*) from maritime sand dunes

**DOI:** 10.1007/s00572-024-01144-w

**Published:** 2024-04-16

**Authors:** Arianna Grassi, Irene Pagliarani, Luciano Avio, Caterina Cristani, Federico Rossi, Alessandra Turrini, Manuela Giovannetti, Monica Agnolucci

**Affiliations:** Department of Agriculture, Food and Environment, University of Pisa, Via del Borghetto 80, 56124 Pisa, Italy

**Keywords:** Arbuscular mycorrhizal symbionts, Coastal sand dunes, Drought stress, Beachgrass, Xerophytic plants

## Abstract

Climate change and global warming have contributed to increase terrestrial drought, causing negative impacts on agricultural production. Drought stress may be addressed using novel agronomic practices and beneficial soil microorganisms, such as arbuscular mycorrhizal fungi (AMF), able to enhance plant use efficiency of soil resources and water and increase plant antioxidant defence systems. Specific traits functional to plant resilience improvement in dry conditions could have developed in AMF growing in association with xerophytic plants in maritime sand dunes, a drought-stressed and low-fertility environment. The most studied of such plants are European beachgrass (*Ammophila arenaria* Link), native to Europe and the Mediterranean basin, and American beachgrass (*Ammophila breviligulata* Fern.), found in North America. Given the critical role of AMF for the survival of these beachgrasses, knowledge of the composition of AMF communities colonizing their roots and rhizospheres and their distribution worldwide is fundamental for the location and isolation of native AMF as potential candidates to be tested for promoting crop growth and resilience under climate change. This review provides quantitative and qualitative data on the occurrence of AMF communities of *A. arenaria* and *A. breviligulata* growing in European, Mediterranean basin and North American maritime sand dunes, as detected by morphological studies, trap culture isolation and molecular methods, and reports on their symbiotic performance. Moreover, the review indicates the dominant AMF species associated with the two *Ammophila* species and the common species to be further studied to assess possible specific traits increasing their host plants resilience toward drought stress under climate change.

## Introduction

Climate change and global warming have contributed to increase terrestrial drought, causing serious negative impacts on agricultural production and posing severe threats to worldwide food security (Dai 2011). Drought is estimated to negatively affect plant growth for more than 50% of arable land by 2050, thus representing an economically and ecologically disruptive event, greatly affecting human life and the world’s food security (Naumann et al. 2021).

Drought stress may be addressed by using novel agronomic practices able to enhance use efficiency of soil natural resources and water and to increase plant antioxidant defence systems. Several studies have been focused on the isolation, selection, and application of beneficial soil microorganisms, such as arbuscular mycorrhizal fungi (AMF), able to enhance drought tolerance in food crops, including cereals, fruit trees, and vegetables, by means of diverse mechanisms beyond improved nutritional status. AMF inoculation may produce changes in root system architecture and functioning and enhance soil water retention in dry sands, thus playing a key role in the performance of plants growing in drought conditions (Augé 2004; Wu et al. 2013; Jayne and Quigley 2014; Pauwels et al. 2023).

Despite the growing recognition of the AMF’s role in plant use efficiency of water and soil resources, the exploitation of specific fungal traits functional to the improvement of plant resilience to climate change remains a significant challenge. Such traits relate to AMF’s ability to germinate, grow, and develop large hyphal networks expressing water and nutrient transporter genes at high temperatures and under drought conditions, as well as to induce the production of antioxidant compounds able to protect plants against oxidative damage caused by abiotic stresses (Rouphael et al. 2015). Another important trait concerns the production of mucilage and exopolysaccharides by AMF and their associated bacteria, compounds which can increase soil aggregation and water retention, thereby helping plants to face drought (Miller and Jastrow 2000; Rillig and Mummey 2006; Püschel et al. 2020; Kakouridis et al. 2022; Pauwels et al. 2023). Moreover, AMF genetic organization represents a further fungal trait possibly affecting mycorrhizal responses, as reported for four potato cultivars colonized by homokaryotic strains, that showed greater host biomass and tuber production, compared with plants inoculated with dikaryotic strains (Terry et al. 2023).

It is conceivable that AMF strains showing the described features may have developed when growing in association with xerophytic plants in maritime sand dunes, a drought-stressed, low-fertility environment for plant growth and development, mainly because of dune instability, low water retention, seasonal extreme temperatures, irradiance, salinity and drought, high evaporation rates, and low concentrations of nutrients and organic matter (Koske and Polson 1984; Maun 2009). Indeed, the survival, establishment, and growth of plants in such unfavourable ecosystems are promoted by AMF, representing an effective means of dune revegetation and restoration, in particular under stress conditions (Sylvia 1989; Gemma and Koske 1997; Corkidi and Rincón 1997; Tadych and Blaszkowski 1999; Gemma et al. 2002; Koske et al. 2004; Camprubi et al. 2011, 2012). Such beneficial effects have been ascribed to extensive extraradical mycelium that functions as an auxiliary absorbing system, with its fine hyphae efficiently exploring the soil and providing host plants with water and mineral nutrients, in particular phosphorus, the most important growth-limiting nutrient in such harsh environments (Read 1989; Giovannetti 2008; Kakouridis et al. 2022; Battini et al. 2016). For example, in maritime sand dunes, AMF mycelium may reach a dry weight of 34 µg per g of sand, representing up to 30% of sand dune microbial biomass (Olsson and Wilhelmsson 2000).

Several studies have been carried out on European beachgrass (*Ammophila arenaria* Link), native to maritime sand dunes of Europe and the Mediterranean basin, and American beachgrass (*Ammophila breviligulata* Fern.), found in North American dunes. These two plant species long have been known as dune-building grasses and recently have been valued for their ecosystem engineering traits (Reijers et al. 2019; Lammers et al. 2023). They are highly mycotrophic species, as reported by many authors worldwide. In North American Atlantic coastal dunes, from Virginia to Maine, *A. breviligulata* represented the dominant sand-colonizing plant species, showing high levels of mycorrhizal colonization, from 20 to 80% of root length (Koske and Polson 1984), while when planted in barren sites in Cape Cod in Massachusetts (USA), 78% of plants were mycorrhizal (Gemma and Koske 1997). In European coastal sand dunes, the percentage of colonized root length of *A. arenaria* ranged from 7–33% in Tuscany (Italy) to 26–80% in the Netherlands, 0–30% in the Northeast of Spain, and 5–70% in six different sites among England, Wales, the Netherlands, Belgium, and Portugal (Giovannetti and Nicolson 1983; Giovannetti 1985; Kowalchuck et al. 2002; Rodríguez-Echeverría et al. 2008; Camprubí et al. 2010). When *A. arenaria* plants were experimentally inoculated with native AMF originating from their rhizosphere, mycorrhizal colonization was 55% (de la Peña et al. 2006).

Given the critical role of the mycorrhizal symbiosis for the survival, nutrition, and growth of these two species of beachgrass living in drought-stressed and low-fertility maritime sand dunes, knowledge of the composition of AMF communities colonizing their roots and rhizospheres is of primary importance because some taxa especially could have developed symbiotic traits aiding the host plants to tolerate such harsh environmental conditions. Accordingly, native AMF from *A. arenaria* and *A. breviligulata* living in maritime sand dunes might be isolated as potential candidates for inocula promoting crop growth and resilience under climate change.

In order to pursue such an objective, in this review we report qualitative and quantitative data on (i) the occurrence of AMF in *A. arenaria* and *A. breviligulata* rhizospheres, as detected by morphological studies and trap culture isolation, in some cases followed by molecular identification; (ii) the richness of AMF communities colonizing beachgrass roots and rhizospheres, as detected by molecular methods, such as PCR cloning and sequencing (PCR-CS), PCR-denaturating gradient gel electrophoresis (PCR-DGGE), and Illumina high-throughput metagenomic sequencing; and on (iii) sand dunes native AMF plant-growth promoting properties.

## Occurrence of AMF in *A. arenaria* and *A. breviligulata* rhizospheres, as detected by morphological studies and trap cultures

**Fig. 1:**
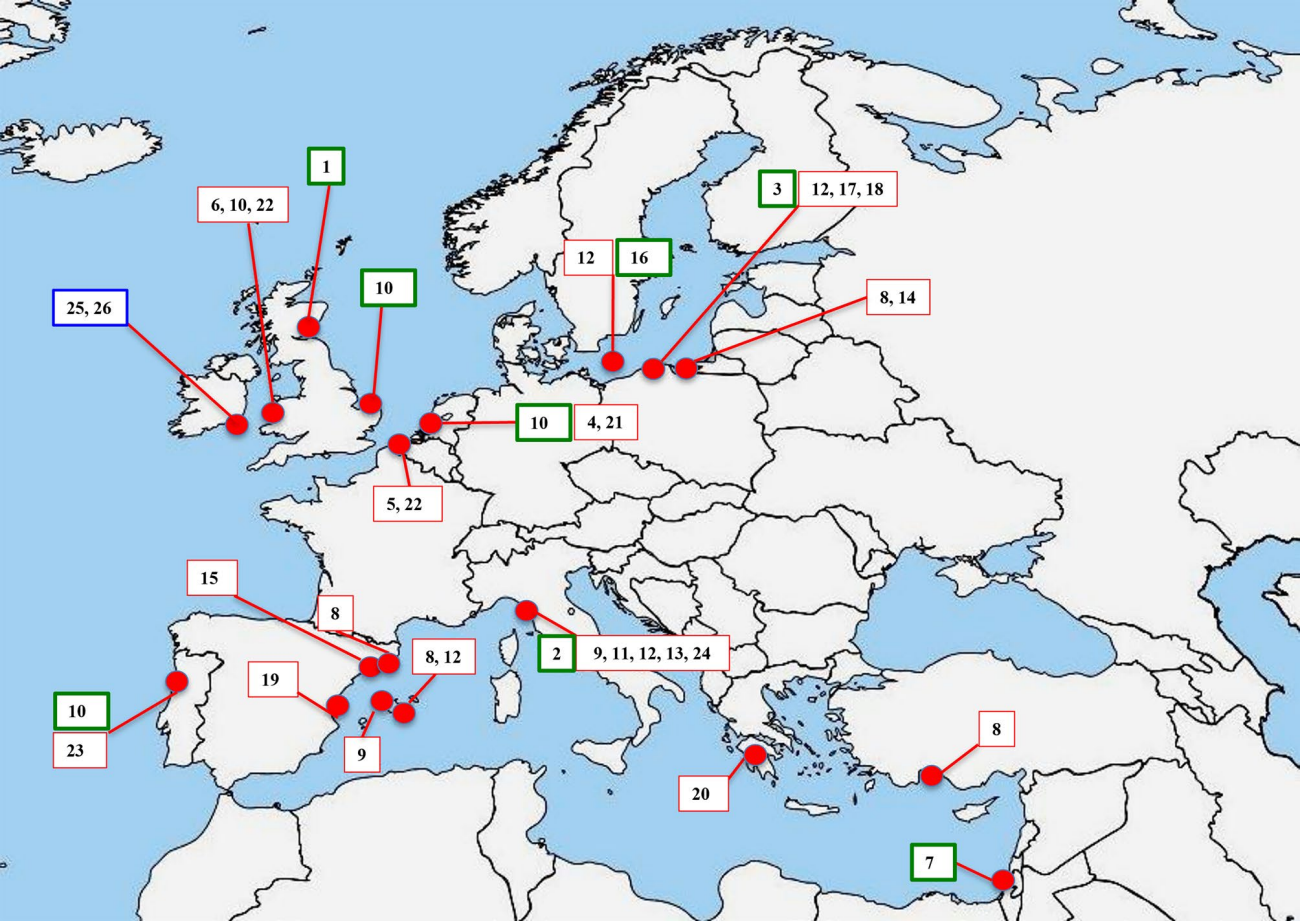
Distribution of the species of arbuscular mycorrhizal fungi associated with *Ammophila arenaria* rhizosphere and roots in maritime sand dunes of Europe and Mediterranean areas. The numbers in the boxes refer to Tables 1 and 2. The different colours of the boxes indicate works based only on morphological studies (green), on morphological studies with taxa confirmed by molecular analyses (red), or only on molecular analyses (blue)

Early evidence of the occurrence of mycorrhizal symbiosis in *A. arenaria* was reported in coastal sand dunes of Scotland and England, UK, with the description of the internal and extraradical mycelium of root endophytes, attributed to *Endogone*, a genus which no longer belongs to the Glomeromycota (Nicolson 1958, 1959, 1960). Successive works confirmed the mycorrhizal status of *A. arenaria* and identified *Rhizoglomus fasciculatum* as its only fungal root symbiont in Scottish sand dunes (Nicolson and Johnston 1979) (Table 1; Fig. 1). Here the generic name *Rhizoglomus* is used, as synonymous with *Rhizophagus* (Sieverding et al. 2015; Walker et al. 2017), and the original names of AMF taxa have been updated following the sites: https://glomeromycota.wixsite.com/lbmicorrizas and http://www.amf-phylogeny.com/.

**Table 1:** Occurrence of AMF in *Ammophila arenaria* and *Ammophila breviligulata* rhizospheres, as detected by morphological studies and trap cultures

**Plant species**	**Spores/100 g (or 100 ml, if indicated) or relative abundance (when % is indicated)**	**AMF species**	**Location**	**References**
*Ammophila arenaria ***(1)**	Spores/100 ml 120–1240	*Rhizoglomus fasciculatum*	Tentsmuir Point Nature Reserve, Scotland, UK	Nicolson and Johnston (1979)
18 sand dunes plant species, including *A. arenaria*, *Helichrysum stoechas, Eryngium maritimum, Medicago marina, medicago littoralis ***(2)**	-	*Funneliformis mosseae*, *Glomus* spp., *Racocetra gregaria, R. fasciculatum, Sclerocystis* spp., *Scutellospora calospora*	Migliarino, Follonica, Alberese, Tuscany, IT	Giovannetti and Nicolson (1983)
*A. arenaria, Agrostis stolonifera, Corynephorus canescens, Festuca rubra* subs. *arenaria Juncus articulates, Juncus balticus ***(3)**	-	21 species, dominated by *Glomus* 107, *Acaulospora koskei, Scutellospora dipurpurescens, Rhizoglomus aggregatum*	Słowiński National Park, Poland	Tadych and Blaszkowski (2000)
*A. arenaria ***(4)**	58–112 25–44	*Glomus* *Scutellospora*	Haringvlietdam and Ouddorp, NL	Kowalchuk et al. (2002)
*A. arenaria ***(5)**	-	*Racocetra castanea, Glomus*	Het Zwin, Knokke-Heist, BE	de la Peña et al. (2006)
*A. arenaria ***(6)**	-	*Glomus*	Ynyslas, Wales, UK	de la Peña et al. (2006)
*A. arenaria ***(7)**	41.4% 38.9% 16.7% 14.8% 14.2% 8.6%	38 species dominated by: *Septoglomus constrictum* *Diversispora aurantia* *Glomus* 178 *S. dipurpurescens* *Funneliformis coronatum* *Diversispora gibbosa*	Kibutz Shefayim to Tel-Aviv, IL	Błaszkowski and Czerniawska (2006)
*A. arenaria ***(8)**	-	*Entrophospora drummondii*	Jurata, Jastrzębia Góra, PL; Cape Salinas, Majorca, ES; Karabucak-Tuzla, TR	Błaszkowski et al. (2006)
*A. arenaria ***(9)**	-	*Entrophospora walkeri*	Cape Salinas, Majorca, ES; Calambrone, Tuscany, IT	Błaszkowski et al. (2006)
*A. arenaria ***(10)**	Spores/50 cm^3^ 16–570	*Acaulospora, Glomus, Scutellospora*	Het Zwin, BE; Oostvoorne, NL; Sao Jacinto and Comporta, PT; Blakeney Point and Ynyslas, UK	Rodríguez-Echeverría et al. (2008)
*A. arenaria ***(11)**	0–68 9–61	*Racocetra fulgida* *Racocetra persica*	UNESCO Biosphere Reserve-Selva Pisana Tuscany, IT	Turrini et al. (2008)
*A. arenaria ***(12)**	-	*Rhizoglomus irregulare*	Bornholm, DK; Calambrone, Tuscany, IT; Słowinski National Park, Poland; El Arenal, Majorca, ES	Błaszkowski et al. (2008)
*A. arenaria ***(13)**	-	*Microkamienskia perpusilla*	Calambrone, Tuscany, IT	Błaszkowski et al. (2009a)
*A. arenaria ***(14)**	-	*Dominikia achra*	Vistula Bar, PL	Błaszkowski et al. (2009b)
*Medicago marina, Lotus creticus, Elymus farctus, Pancratium maritimum, Calystegia soldanella, Ammophila arenaria ***(15)**	2–77	*Glomus ambisporum, Rhizoglomus clarum, Oehlia diaphana, Rhizoglomus intraradices, Rhizoglomus microaggregatum*, *R. persica*	Les Salines, ES	Camprubí et al. (2010)
*A. arenaria ***(16)**		32 species, dominated by:	Bornholm Island, DK	Błaszkowski and Czerniawska (2011)
	73.6%	*R. irregulare*		
	19.4%	*S. dipurpurescens*		
	10.3%	*Archaeospora trappei*		
	9.0%	*R. aggregatum*		
	7.1%	*Diversispora eburnea*		
	6.5%	*Scutellospora armeniaca*		
	5.2%	*Paraglomus laccatum*		
*A. arenaria*, *Carex arenaria, Corynephorus canescens, Juncus articulatus ***(17)**	-	*Glomus tetrastratosum*	Słowinski National Park, PL	Błaszkowski et al. (2014a)
*A. arenaria ***(18)**	-	*Diversispora peridiata, Diversispora slowinskiensis*	Słowinski National Park, PL	Błaszkowski et al. (2015)
*A. arenaria ***(19)**	-	*Diversispora valentina*	Gulf of Valencia, ES	Guillén et al. (2020a)
*A. arenaria ***(20)**	-	*Complexispora multistratosa, Complexispora mediterranea*	Voidokoilia, Peloponnese Peninsula, EL	Błaszkowski et al. (2023)
*Ammophila breviligulata*	139 96 5 4 0.3	*Acaulospora scrobiculata* *Gigaspora gigantea* *Entrophospora etunicata* *S. calospora* *Gigaspora* sp.	Moonstone Beach, Rhode Island, USA	Koske (1981)
*A. breviligulata*	27–198 5-338 0–13 0–9 0–1	*G. gigantea* *A. scrobiculata* *S. calospora* *E. etunicata* *Gigaspora* sp.	South Kingstown, Rhode Island, USA	Koske and Halvorson (1981)
*A. breviligulata, Lathyrus japonicus*, *Solidago sempervirens*	-	*Quatunica erythropa*	Moonstone Beach, RI, USA	Koske and Walker (1984)
*A. breviligulata*	16.2 5.2 1.7 0.5 0.2	*G. gigantea* *R. persica* *S. calospora* *Q. erythropa* *Cetraspora pellucida*	Cape Cod, MA, USA	Bergen and Koske (1984)
*A. breviligulata*	77%	*A. scrobiculata*	Moonstone Beach, RI, USA	Tews and Koske (1986)
	67%	*G. gigantea*		
	50%	*R. persica*		
	33%	*R. fasciculatum*		
	30%	*Diversispora pustulata*		
	30%	*Q. erythropa*		
	20%	*S. calospora*		
	17%	*R. aggregatum*		
	3%	*Paraglomus occultum*		
*A. breviligulata*	6.9–12.2 1.6–2.5 0.5–3.7 0.6–2.9 0.9–1.3	*R. persica* *A. scrobiculata* *G. gigantea* *S. calospora* *Q. erythropa*	Moonstone Beach, RI, USA	Friese and Koske (1991)
Sand dune plants dominated by *A. breviligulata*	-	*Acaulospora lacunosa, Acaulospora mellea, A. scrobiculata, Acaulospora* spp., *C. pellucida*, *Dentiscutata reticulata*, *Entrophospora infrequens, G. gigantea, Gigaspora margarita, Glomus* spp., *Q. erythropa, R. persica, R. clarum, R. microaggregatum, S. calospora*	Cape Cod, Provincetown, MA, USA	Koske and Gemma (1997)
*A. breviligulata*	Spores/100 ml 47 1 0.5	*G. margarita* *R. clarum* *S. calospora*	Cape Cod, MA, USA	Gemma and Koske (1997)
*A. breviligulata*	-	34 species dominated by *R. irregulare, C. tortuosum, R. aggregatum, F. mosseae, R. microaggregatum, S. calospora, Funneliformis geosporum, A. lacunosa*	Magdalen Islands archipelago, Québec, Canada	Dalpé et al. (2016)

The names of AMF taxa refer to the current names of each species reported in the original works

In the column “Plant species”, the numbers in parentheses refer to the studies shown in Fig. 1

**Table 2:** Occurrence of AMF communities in *Ammophila arenaria* and *Ammophila breviligulata* rhizosphere and roots, as detected by molecular methods

**Plant species**	**Molecular analysis/primers**	**AMF species**	**Location**	**References**
*Ammophila arenaria ***(21)**	DNA extraction from roots and spores, PCR-DGGE and band sequencing/NS31-AM1 (Simon et al. 1992; Helgason et al. 1998)	*Cetraspora pellucida, Dentiscutata cerradensis, Diversispora spurca, Funneliformis coronatum, Funneliformis fragilistratum, Glomus, Racocetra castanea*	Haringvlietdam and Ouddorp, NL	Kowalchuk et al. (2002)
*A. arenaria ***(22)**	DNA extraction from roots, cloning and sequencing/NS31-AM1	*Glomus, Rhizoglomus fasciculatum*, *Rhizoglomus intraradices*, *Rhizoglomus vesiculiferum*	Het Zwin, Knokke-Heist, BE; Ynyslas, Wales, UK	de la Peña et al. (2006)
*A. arenaria ***(23)**	DNA extraction from spores and roots, cloning and sequencing/NS31-AM1	*Corymbiglomus globiferum, Racocetra persica, R. intraradices, R. fasciculatum, Septoglomus constrictum, Simiglomus hoi*	Natural Reserve of São Jacinto and Comporta, PT	Rodríguez-Echeverría and Freitas (2006)
*A. arenaria Helichrysum stoechas, Otanthus maritimus ***(24)**	DNA extraction from spores, cloning and sequencing/SSU-1f - AM1- (Turrini et al. 2008) and ITS1 and ITS4 (Gardes and Bruns 1993).	*Racocetra fulgida, Racocetra persica*	UNESCO Biosphere Reserve-Selva Pisana Tuscany, IT	Turrini et al. (2008)
*A. arenaria ***(25)**	DNA extraction from roots and Illumina Miseq sequencing/WANDA and AML2 (Dumbrell et al. 2011; Lee et al. 2008)	*Entrophospora, Glomus, Paraglomus, Gigasporaceae*	Curracloe, Co. Wexford, IE	Lastovetsky et al. (2022)
*A. arenaria ***(26)**	DNA extraction from spores, PCR amplification and sequencing/WANDA and AML2 (Dumbrell et al. 2011; Lee et al. 2008)	*Acaulospora, Ambispora, Diversispora, Funneliformis, Glomus, Paraglomus, Scutellospora*	Curracloe, Co. Wexford, IE	Lastovetsky et al. (2024)
*Ammophila breviligulata*	DNA extraction from spores, PCR amplification and sequencing/LR1 and NDL22	*Acaulospora, Cetraspora, Corymbiglomus, Dentiscutata, Diversispora, Gigaspora gigantea, Gigaspora albida, Gigaspora rosea, Racocetra, Scutellospora*	Cape Cod, MA, USA	Lastovetsky et al. (2018)

In the column “Plant species”, the numbers in parentheses refer to the studies shown in Fig. 1

Successive studies after 1979 found many AMF species from *A. arenaria* rhizospheres, regardless of geographic location. For example, 25 different AMF species were described from maritime sand dunes adjacent to the Mediterranean Sea in Israeli (Błaszkowski and Czerniawska 2006), and 21 and 26 from dunes adjacent to the Baltic Sea in Słowiński National Park (Poland) and in Bornholm Island (Denmark), respectively (Tadych and Błaszkowski 2000; Błaszkowski and Czerniawska 2011) (Table 1; Fig. 1).

It is interesting to note that several new species from the rhizosphere of *A. arenaria* growing in maritime sand dunes were isolated and/or molecularly described: *Complexispora multistratosa*, *Complexispora mediterranea*, *Dominikia achra*, *Diversispora peridiata*, *Diversispora slowinskiensis*, *Diversispora valentine*, *Entrophospora drummondii*, *Glomus tetrastratosum*, *Microkamienskia perpusilla*, *Rhizoglomus irregulare*, *Septoglomus jasnowskae* (Błaszkowski et al. 2006, 2008, 2009a, b, 2014a, b, 2015, 2023; Guillén et al. 2020a, b) (Table 1; Fig. 1).

**Fig. 2:**
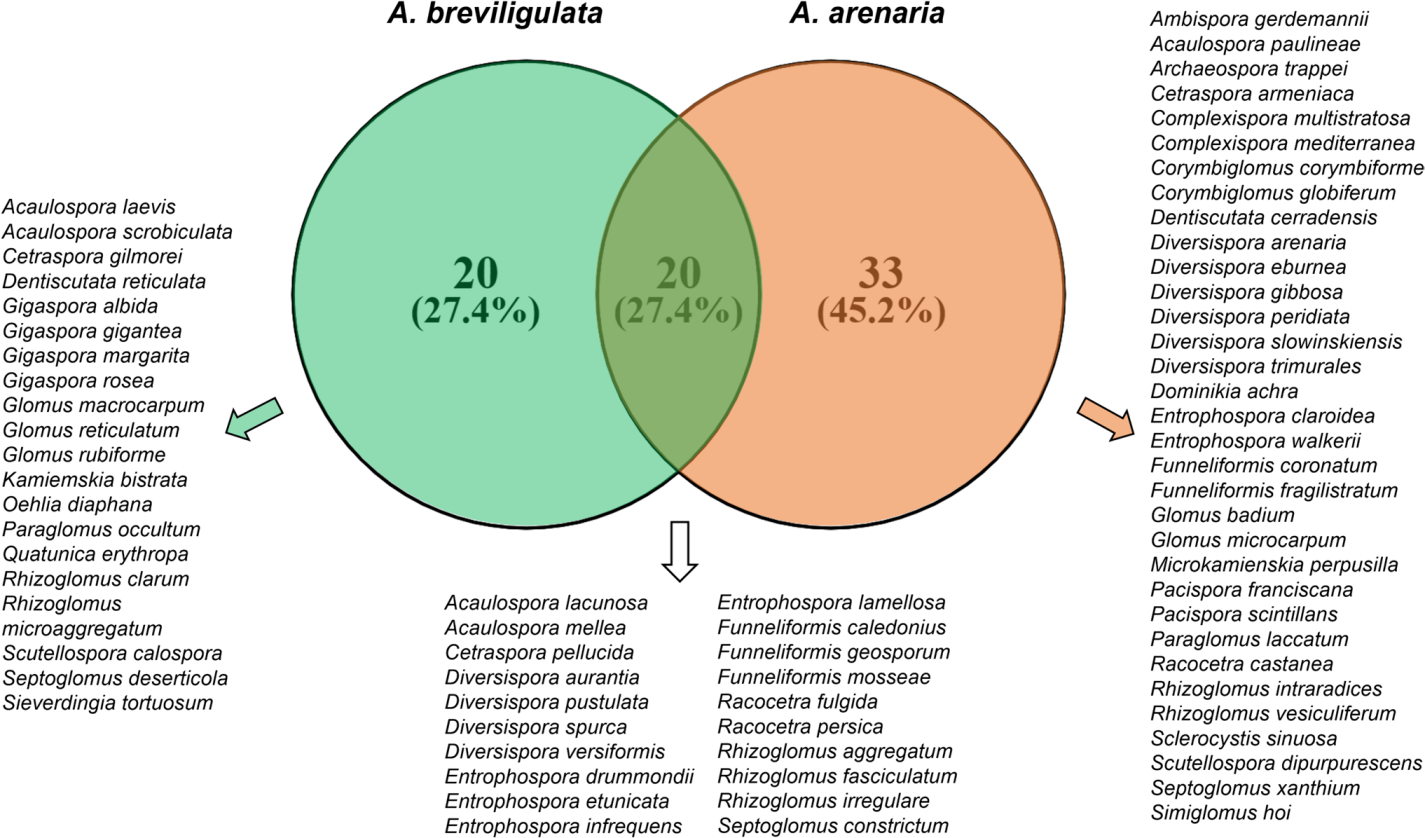
Venn diagram showing the AMF species associated with *Ammophila arenaria* (green), *Ammophila breviligulata* (orange), and shared taxa

On the other side of the Atlantic Ocean, only AMF species associated with *A. breviligulata* were investigated, while no studies were performed on AMF species of *A. arenaria* in the west coast of the USA, where it is considered an invasive species. The data on *A. breviligulata* come from only three places in North Atlantic sand dunes, with high numbers of AMF species recovered, 17 and 29 from Cape Cod, MA and the Magdalen Islands archipelago, Québec, respectively (Koske and Gemma 1997; Dalpé et al. 2016) (Table 1). It is interesting to note that in Rhode Island and Cape Cod sand dunes, *A. breviligulata* was predominantly associated with *Acaulospora scrobiculata* and *Gigaspora gigantea* (Koske 1981; Koske and Halvorson 1981), two species that were not found in *A. arenaria* rhizospheres (Fig. 2). Only one new AMF species associated with *A. breviligulata* was described, *Quatunica erythropa* (Koske and Walker 1984), probably because of few studies carried out on such species (10 studies), compared with those on *A. arenaria* (26) and to the absence of studies in the years from 1997 to 2016 (Table 1).

## Occurrence of AMF communities colonizing *A. arenaria* and *A. breviligulata* roots, as detected by molecular methods

The first thorough molecular study of an AMF community associated with *A. arenaria* was performed by Kowalchuk et al. (2002) in Dutch coastal sand dunes, utilizing the polymerase chain reaction-denaturing gradient gel electrophoresis (PCR-DGGE) targeting the 18S rRNA gene (using the AM1 and NS31-GC clamp primer pair). Sequence analysis of excised DGGE bands allowed the detection of at least seven different species, belonging to the genera *Glomus* and *Scutellospora*, i.e. *F. coronatum, F. fragilistratus*, *Diversispora spurca*, *Glomus* sp., *Racocetra castanea*, *Dentiscutata cerradensis*, and *C. pellucida*. Although PCR-DGGE is an excellent molecular method able to distinguish even minor levels of sequence variation, AMF species occurring in low abundance were overlooked, such as *Acaulospora* and *Glomus* spp. of which a few spores were isolated from sand, while their sequences were not recovered from DGGE bands (Table 2). This could be ascribed to the method which allows the detection of populations representing > 1–2% of the total and also ascribed to the primer pair used, unable to provide total coverage of the AMF clade (Redecker et al. 2000).

The richness and composition of AMF communities associated with *A. arenaria* in Belgium coastal sand dunes, reproduced in trap cultures and investigated by PCR cloning and sequencing using the primers NS31/AM1, produced 31 sequences of the genus *Glomus*, while no *Scutellospora* sequences were recovered, although detected in the rhizosphere by morphological observations (de la Peña et al. 2006). This confirms the difficulty of covering the entire AMF clade by the primers used. The same primers, utilized for cloning and sequencing a fragment of the SSU rDNA extracted and amplified from the roots of *A. arenaria* in Portuguese sand dunes, allowed the detection of AMF sequences belonging to the genus *Glomus*, some of which clustered with *Rhizoglomus intraradices*, *R. fasciculatum*, and *Septoglomus constrictum* (Rodríguez-Echeverría and Freitas 2006). It is interesting to note that the sequencing of spores obtained from trap cultures showed the presence of *Racocetra persica*, whose sequences were not retrieved from *A. arenaria* roots, confirming previous findings and suggesting that the low level of root colonization by *R. persica* could have led to a preferential amplification of the more abundant sequences of *Glomus* spp. during PCR (Rodríguez-Echeverría and Freitas 2006) (Table 2).

The regular occurrence of *Racocetra fulgida* and *Racocetra persica* in *A. arenaria* rhizospheres, as assessed by morphological methods, was confirmed utilizing both NS31/AM1 and ITS1F/ITS4 primers in an in situ collection of coastal sand dunes AMF within a UNESCO Biosphere Reserve in Tuscany, Italy (Turrini et al. 2008). Two recent studies on sand dunes systems located at Curracloe, Wexford, Ireland, showed that the AMF root communities of *A. arenaria* comprised *Gigasporaceae, Entrophospora*, *Paraglomus, *and *Glomus*, while AMF spores collected from the rhizosphere showed a greater richness, although a taxonomic assignment to the species level was not provided (Lastovetsky et al. 2022, 2024) (Table 2).

The only molecular work investigating AMF diversity in the rhizosphere of *A. breviligulata* reported the occurrence of *G. gigantea*, *G. albida, G. rosea*, *Racocetra* spp., *Scutellospora* spp., *Cetraspora* sp., *Acaulospora* spp., *Dentiscutata* sp., and *Corymbiglomus* sp. in plants growing in North Atlantic maritime sand dunes at Cape Cod National Seashore, MA, USA (Lastovetsky et al. 2018).

It is interesting to note that different AMF species were associated with *A. arenaria* and *A. breviligulata*: only 20 species in common have been reported, while 20 were recovered only from *A. breviligulata* and 33 only from *A. arenaria* (Fig. 2). The most surprising finding is represented by the consistent occurrence of taxa of the genus *Gigaspora* from *A. breviligulata*, i.e. *G. albida*, *G. gigantea*, *G. margarita, *and *G. rosea*, taxa that were never recovered from *A. arenaria*, although two of them, *G. gigantea* and *G. margarita*, occurred in sand dunes of the Paleartic biogeographical realm (Sturmer et al. 2018). It is possible that such species either did not occur in the investigated sites or showed a selective association with plants other than *A. arenaria*.

## Symbiotic performance of native sand dune AMF

The hypothesis that AMF isolated from maritime sand dunes might have developed symbiotic traits functional to plant survival and growth in such a harsh environment stimulated a few studies, aimed at assessing the performance of native AMF isolated from the rhizosphere of sand dune plants. As early as 1979, Nicolson and Johnston carried out the first plant growth experiment in pots, using unsterile dune sands, utilizing *R. fasciculatum* from Scottish maritime sand dunes, as compared with *F. geosporum* from agricultural soils, inoculated on *A. arenaria* and *Zea mays*. Both AMF significantly improved *A. arenaria* growth, compared with control plants: in young *A. arenaria* plants dry weight increased by 18%, while maize plants dry weights were not significantly different when inoculated with *R. fasciculatum* or *F. geosporum* alone, showing significant increases - up to 120% - only when the two AMF were inoculated together (Nicolson and Johnston 1979). Although the authors concluded “that plants grow in such adverse conditions as sand dunes because they are mycorrhizal”, control plants were able to grow, even if poorly.

In a revegetation and restoration program of a dune system where naturally occurring plants previously had been destroyed by grazing livestock and human use, in Cape Cod National Seashore, MA, USA, the inoculation of *A. breviligulata* with the dominant native AMF *G. margarita*, *R. clarum* and *S. calospora*, produced significant increases in culms (+14%) and inflorescences (+67%), compared with control plants (Gemma and Koske 1997).

The mixture of two AMF, *Septoglomus deserticola* and *Glomus macrocarpum*, isolated from sand dunes along the north Atlantic coast of Florida increased shoot dry weight, root length, and plant height by 219, 81, and 64%, respectively, compared with control plants, in a replenishment study with the sand dune grass *Uniola paniculata* (sea oats) at Miami Beach, Florida (Sylvia 1989). The higher performance of sand dunes native AMF, as compared with two different commercial isolates, was reported in a study performed in Iceland on another sand dune grass, *Leymus arenarius*; interestingly, the foliage and root dry mass were the highest when inoculated with native AMF even compared with added phosphorus treatments (Greipsson and El-Mayas 2000). Similarly, native AMF isolated from Het Zwin, Knokke-Heist natural reserve, in Belgium, significantly increased biomass (60%), number of tillers (45%), and leaves (26%) in young *A. arenaria* plants while reducing root infection and multiplication of the root-feeding nematode *Pratylenchus penetrans* (de la Peña et al. 2006).

A microcosm experiment carried out at the University of Rhode Island, USA, showed that native AMF inoculum significantly increased the survival ability of *A. breviligulata* in dune sand under drought stress, as 78% of mycorrhizal plants survived, against 20% of non-inoculated ones (Koske and Polson 1984). As to saline stress, AMF isolates from coastal sand dunes did not enhance lettuce leaf biomass, compared with isolates originating from desert or field soil (Tigka and Ipsilantis 2020), but three out of six AMF assemblages from Greek coastal sand dunes under high salinity levels helped olive tree cuttings to tolerate the stress (Kavroulakis et al. 2020).

An interesting work was carried out on 15 sand dune plant species that comprised 12 grasses and three shrubs (Camprubi et al. 2011). The authors assessed the growth effects of a consortium of native AMF isolated from six sand dune plants including *A. arenaria* in the Mediterranean northeast coast of Spain as compared with no inoculation and with *R. intraradices* BEG72, a strain isolated from an agricultural soil in Spain that had been shown to be highly efficient in a wide range of experimental studies. They reported that seven plant species showed higher dry weights when inoculated with either BEG72 or the native AMF versus the controls, while five of those plant species increased more when inoculated with the native AMF than with BEG72. For example, *Otanthus maritimus* and *Elymus farctus* growth was significantly improved by native AMF but not by BEG72, although the reverse was also found, as only BEG72 boosted *Ononis natrix* and *Armeria maritima* growth. Moreover, the study revealed a high mycorrhizal dependency of five of the plant species investigated, suggesting that, overall, the limited growth of control plants could be ascribed to the lack of adequate AMF inoculum (Camprubi et al. 2011). As the consortium of native AMF was composed of diverse AMF species, it is conceivable that they reflected a high functional diversity, consistent with previous findings (Munkvold et al. 2004; Mensah et al. 2015; Turrini et al. 2018).

Overall, the few works performed to test the symbiotic performance of AMF isolated from sand dunes did not investigate the mechanistic aspects of AMF activity, which could uncover the potentially beneficial fungal traits relevant to drought tolerance. Hence, the question as to whether AMF isolated from the rhizospheres of maritime sand dune plants may promote plant growth and resilience, protecting agricultural plants from drought, represents a demanding challenge to be pursued by further extensive and in-depth studies.

## Concluding remarks and future perspectives

This review shows that *A. arenaria* and *A. breviligulata* growing in the harsh environment of maritime sand dunes, subject to the selective pressure of seasonal extreme temperatures and drought, host rich and large AMF communities in their roots and rhizospheres. Qualitative and quantitative data are provided on the occurrence of the diverse AMF species in different sites in Europe, the Mediterranean basin, the USA, and Canada. The dominant species belong to Gigasporales and Glomerales, consistent with previous data on their prevalence in maritime sand dunes worldwide (Stürmer et al. 2018), but members of Acaulosporaceae and Diversisporaceae also are present.

Among the 33 AMF species found with *A. arenaria*, the most frequently recovered are those belonging to the group *Rhizoglomus fasciculatum/intraradices/irregulare*, occurring in the sand dunes of several countries across Europe and the Mediterranean basin, i.e. Belgium, Denmark, Italy, Poland, Portugal, Spain, and the UK, together with *Racocetra persica* occurring in Italy, Spain, and Portugal, and *Funneliformis coronatum* found in the Netherlands and Israel. Among the 20 AMF species recovered from *A. breviligulata*, *Acaulospora scrobiculata*, and *Gigaspoa gigantea* are the most frequent, while *R. persica* was the prevalent species among those common to the two plant species. Such fungus species could be further studied to assess possible specific traits allowing their host plants to withstand environmental stresses and thrive in hostile ecosystems. As there has been very little testing of AMF isolates from maritime sand dunes with crop plants under drought-stressed agronomic conditions, further investigations should be carried out in microcosms, macrocosms and in the field, under different levels of drought stress, in order to assess the ability of such AMF isolates to survive in the new soil environment and compete with native symbionts, while maintaining their potential beneficial traits.

In the years to come, the availability of AMF showing promising beneficial characteristics will allow the formulation of innovative consortia, to be commercially reproduced and utilized as a viable alternative or in addition to current ones. Such newly designed consortia could be used as inoculants for increasing plant water and nutrient use efficiency, in turn enhancing crop productivity and resilience toward drought stress in sustainable food production systems under climate change.

## Data Availability

No datasets were generated or analysed during the current study.
